# Highlighted multi-modifications of enzymes: a novel succinylation mediated by histone acetyltransferase 1 in tumors

**DOI:** 10.20892/j.issn.2095-3941.2021.0533

**Published:** 2021-12-22

**Authors:** Xiaodong Zhang, Chunyu Hou, Guang Yang

**Affiliations:** 1 Department of Gastrointestinal Cancer Biology, Liver Cancer Center, Tianjin Medical University Cancer Institute & Hospital, National Clinical Research Center for Cancer, Key Laboratory of Cancer Prevention and Therapy, Tianjin, Tianjin’s Clinical Research Center for Cancer, Tianjin 300060, China

## Histone acetyltransferase 1 serves as a succinyl transferase in tumors

Protein post-translational modification (PTM) as an important research field has attracted increasing attention. Recently, our group has reported that histone acetyltransferase 1 (HAT1) succinylated histones and non-histones and promoted tumorigenesis^1^. HAT1 plays a role in different biological processes, including cell cycle progression, glucose metabolism, histone production, and DNA damage repair^2^. We found that HAT1 succinylated histone H3 on K122. For a non-histone, HAT1 catalyzed the succinylation of phosphoglycerate mutase 1 (PGAM1) on K99 to increase its enzyme activity, promoting glycolysis in cancer cells. Functionally, HAT1-mediated succinylation contributed to tumorigenesis. Our finding provided new insight into the mechanism of how a histone acetyltransferase HAT1 modulated succinylation during cancer development.

## Highlighted multi-modifications mediated by a single enzyme

PTMs, which are alterations in the amino acid sequence of a protein after its synthesis, increase the functional diversity of the proteome by the covalent addition of functional groups or proteins, proteolytic cleavage of regulatory subunits, and degradation of entire proteins, which may be involved in the modification of amino acid side chains, terminal amino acids, or carboxyl groups by means of covalent or enzymatic modifications following protein biosynthesis^3^. Generally, these modifications influence the structure, stability, activity, cellular localization, and substrate specificities of proteins. In this study, we highlighted the significance of PTMs in tumors.

PTMs confer complexity to the proteome by adding diverse functions using a limiting the number of genes^3^. PTMs participate in the regulation of gene expression, modifying protein functions, and modulating interactions of proteins with DNA, cofactors, lipids, and other proteins, involving protein phosphorylation, protein methylation, and protein acetylation. Phosphorylation is an important PTM for modulating protein functions. The transfer of phosphate groups to the amino acid residues (serine, threonine, and tyrosine) of proteins catalyzed by protein kinases, and the binding of GTP under the action of signals, are common regulatory modes, which play important roles in the process of cell signal transduction^4^. Methylation is a covalent modification of arginine and lysine. Arginine can undergo monomethylation or dimethylation, while lysine can undergo monomethylation, dimethylation, or trimethylation^5^. Glycosylation is the process of attaching sugars to proteins or lipids under the control of enzymes, beginning in the endoplasmic reticulum and ending in the Golgi apparatus. Glycosyltransferases transfer sugars to proteins, forming glycosidic bonds with amino acid residues on proteins^6^. Sumoylation is analogous to ubiquitinylation in terms of the reaction scheme and enzyme classes. Sumoylation results in the addition of SUMOs (small ubiquitin-like modifiers), affecting the structure and subcellular localizations of proteins^7^. Hydroxylation attaches a hydroxylgroup (-OH) to a side chain of a protein, and is the most common reaction type in phase I metabolism. It usually produces a chemically stable and more polar hydroxylated metabolite than a drug^8^. Lysine succinylation (Ksuc or Ksucc) is a newly identified histone PTM that changes the chemical environment of histones and is similar to other acylation modifications. Lysine succinylation appears to accumulate at transcriptional start sites and to correlate with gene expression. Importantly, lysine succinylation plays an important role in cancers. In gastric cancer, overexpression of a succinylation-mimic mutant of lactate dehydrogenase A promotes cell proliferation, invasion, and migration^9^.

With the in-depth study of PTMs, it has been reported that an enzyme has a variety of modification functions, such as P300 as a transcriptional co-activator modulating the gene transcription through acylation of histones^4,10^. P300 is able to catalyze multiple types of lysine PTMs, such as acetylation, crotonylation, and lysine 2-hydroxyisobutyrylation^11^. HAT1 also has multiple functions, involving acetyltransferase, isobutyryltransferase, and succinylation activities on both histones and/or non-histones^2,12–14^. Nucleosomes are comprised of a core histone coatomer (2 copies each of histones H2A, H2B, H3, and H4), enclosed by ~147 bases of DNA. Whereas histone protein isoforms exist for H2A, H2B, and H3 (e.g., macro-H2A and cenH3), there are no histone H4 protein isoforms in animals^2^, indicating that H4 is a core subunit of all nucleosomes and may therefore represent a key focal point for regulation. For example, newly synthesized histone H4 is di-acetylated on lysines 5 and 12 by the cytosolic HAT1^15^. For non-histones, HAT1 moderates the NF-κB response by regulating the transcription factor, PLZF^12^. Considering that HAT1 is an enzyme with multi-modification abilities, we predict that other enzymes may also involve multi-modifications in tumors. Based on its multiple regulatory functions, HAT1 may therefore serve as a precise therapeutic target in tumors.

## Perspectives

### Reprograming of protein PTMs

In recent years, with extensive studies of protein PTMs, the following research topics have arisen. Importantly, reprogramming of PTMs is observed in tumors. Accordingly, there is increased interest in the reprogramming of protein PTMs in tumors. What happens in tumors during reprogramming of PTMs? How do multi-modification enzymes function in tumors? What are the structural characteristics of enzymes with multiple catalytic activities? What are the relationships among these different catalytic functions? Does the function of catalytic pockets depend on its substrates? How do the various modification functions of enzymes play a coordinating role in tumors? To answer these questions, increasing studies should provide new insights into the mechanism by which the reprogramming of PTMs functions in tumors.

### Enzymes with multi-modifications are ideal drug targets

PTMs are involved in many precise regulation mechanisms, playing crucial roles in cellular physiological and pathological processes^16^. However, the significance of PTMs in the development of cancer is poorly understood. Enzyme-mediated multi-modifications have a global influence in cancer progression. Notably, the multi-modifications mediated by enzymes in tumors, such as the P300 and HAT1 enzymes, should be extensively studied in the future. Furthermore, the network of multi-modifications mediated by one enzyme needs to be demonstrated in detail. The results may show that enzymes with multiple regulatory functions can serve as ideal drug targets in the treatment of tumors.
